# Neuronal circuits and physiological roles of the basal ganglia in terms of transmitters, receptors and related disorders

**DOI:** 10.1007/s12576-016-0445-4

**Published:** 2016-03-15

**Authors:** Katsuya Yamada, Susumu Takahashi, Fuyuki Karube, Fumino Fujiyama, Kazuto Kobayashi, Akinori Nishi, Toshihiko Momiyama

**Affiliations:** 1 Department of Physiology, Hirosaki University Graduate School of Medicine, Hirosaki, Japan; 2 Laboratory of Neural Circuitry, Graduate School of Brain Science, Doshisha University, Kyotanabe, Japan; 3 Department of Molecular Genetics, Fukushima Medical University, Fukushima, Japan; 4 Department of Pharmacology, Kurume University School of Medicine, Kurume, Japan; 5 Department of Pharmacology, Jikei University School of Medicine, Nishi-Shinbashi Campus, Minato-ku, Tokyo, 105-8461 Japan

**Keywords:** Striatum, Parkinson’s disease, Deep brain stimulation, Closed-loop control, Optogenetic technology

## Abstract

The authors have reviewed recent research advances in basal ganglia circuitry and function, as well as in related disorders from multidisciplinary perspectives derived from the results of morphological, electrophysiological, behavioral, biochemical and molecular biological studies. Based on their expertise in their respective fields, as denoted in the text, the authors discuss five distinct research topics, as follows: (1) area-specific dopamine receptor expression of astrocytes in basal ganglia, (2) the role of physiologically released dopamine in the striatum, (3) control of behavioral flexibility by striatal cholinergic interneurons, (4) regulation of phosphorylation states of DARPP-32 by protein phosphatases and (5) physiological perspective on deep brain stimulation with optogenetics and closed-loop control for ameliorating parkinsonism.

## Introduction

Basal ganglia (BG) are a complex network of nuclei in the forebrain which play critical roles in motor control. It has been suggested that any damage to/disorganization of the BG may be closely related to various neurodegenerative diseases, such as Parkinson’s disease (PD) [1]. The roles of BG can be envisioned those of processing information streams through several neuronal circuits composed of a variety of neurons as well as glial cells [2]. Although the profiles of these neurons have been clarified [3], detailed knowledge of the transmitters, modulators and the respective receptors involved in these functional circuits is currently limited.

Dopamine (DA) is one of the critical neurotransmitters and/or neuromodulators in BG circuitries, affecting the control of motor activity and emotion as well as abuses of addictive drugs [4]. Dopaminergic neurons in the substantia nigra pars compacta project their axons towards medium spiny neurons and cholinergic interneurons in the striatum [5, 6], thereby regulating the neuronal activities of these striatal neurons. The nigro-striatal dopaminergic pathway has important functions in motor control [7] through the interaction DA and acetylcholine (ACh) [8, 9]. Although recent molecular biological, biochemical, pharmacological and electrophysiological studies have revealed the profiles of DA receptors [10], little information is yet available on the mechanisms of DA release, action of physiologically released DA or the regulatory roles of these receptors in brain functions.

In this review, recent findings on the BG circuitry and function are presented and discussed by experts in the field of BG research, based on studies which used refined tools for morphology, electrophysiology, biochemistry and molecular biology. These findings may provide a clue to the understanding of new aspects of BG functions, opening the doors to new strategies for the therapeutics of BG-related disorders.

## Area-specific DA receptor expression of astrocytes in basal ganglia (Katsuya Yamada)

**Fig. 1:**
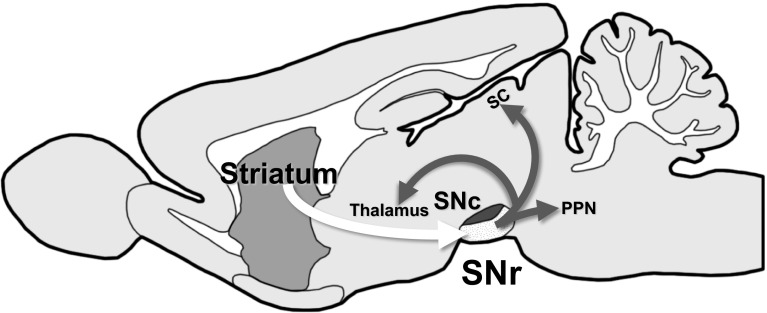
Schematic diagram of information flow through striatonigral axis. *SNr* Substantia nigra pars reticulata, *SNc* substantia nigra pars compacta, *SC* superior colliculus, *PPN* pedunculopontine nucleus

The substantia nigra pars reticulata (SNr), a nucleus located in the midbrain and a major output nucleus of the basal ganglia, consists mostly of gamma-aminobutyric acid-ergic (GABAergic) neurons. These SNr GABAergic neurons receive inputs from the striatum and project their axons to remote nuclei, such as the superior colliculus, thalamus and pedunculopontine nucleus of the brain stem (Fig. 1). One of the physiological roles of SNr is to regulate motor activity depending upon information processed in the striatum [11]. The SNr may also act as a sensor of hypoxic/hypoglycemic conditions [12–14].

The nucleus adjacent to the SNr is the substantia nigra pars compacta (SNc), consisting mainly of dopaminergic neurons. It is the selective loss of SNc neurons which is a major cause of Parkinson’s disease. Interestingly, it has been well established that SNc dopaminergic neurons release dopamine from their dendrite which extends deeply into the SNr (dendritic release) [15]. SNr cells targeted by the dendritically released dopamine are not yet fully understood and unlike axonal release, non-synaptic release from the dendrites makes it difficult to identify the target cells.

SNr GABAergic neurons show high-frequency spontaneous firings that can be recorded in acute slices and even in acutely dissociated neurons, providing valuable information [12–14]. Based on our own experience using such acute slices and single cells in SNr, it seems unlikely that dopamine directly affects SNr GABAergic neuron firing.

 Immunohistochemistry studies have shown that SNr mainly expresses DA D1 receptors (D1R), whereas SNc abundantly expresses dopamine D2 receptors (D2R) [16]. Thus, the cells being targeted by the dendritically released dopamine may well express D1R. Although it is widely accepted that D1R is functionally expressed on the striatonigral axons [17], the very dense immunoreactivity pattern for D1R in the SNr led us to explore whether cellular components other than neurons are involved in the expression as well. However, due to extremely fine D1R immunoreactivity in the SNr, our initial confocal microscopic examination of SNr slices using antibodies, such as against D1R/Parvalbumin, D1R/tyrosine hydroxylase, D1R/glial fibrillary acidic protein and D1R/3-phospho-d-glycerate dehydrogenase, did not provide conclusive evidence of the involvement of other cellular components. Alternatively, Katsuhiro Nagatomo from our laboratory has successfully utilized D1R promoter-controlled yellow fluorescent protein-expressing transgenic mouse provided by Prof. Kazuto Kobayashi to identify the cellular component expressing D1R. Combined with information obtained from double immunocytochemistry studies, we also confirmed that the heterogeneous D1R expression in astrocytes is not restricted to the SNr but also appears more widely in the BG.

In PD patients, a decrease in the number of SNc neuron dendrites might well reduce dopamine-mediated, non-striatonigral regulation of SNr function related to motor movement and/or sensing energy status. It may be of interest to investigate how the dendritically released dopamine influences neurons/glia interplay in the SNr circuitry.

## The role of physiologically released DA in the striatum (Toshihiko Momiyama)

**Fig. 2:**
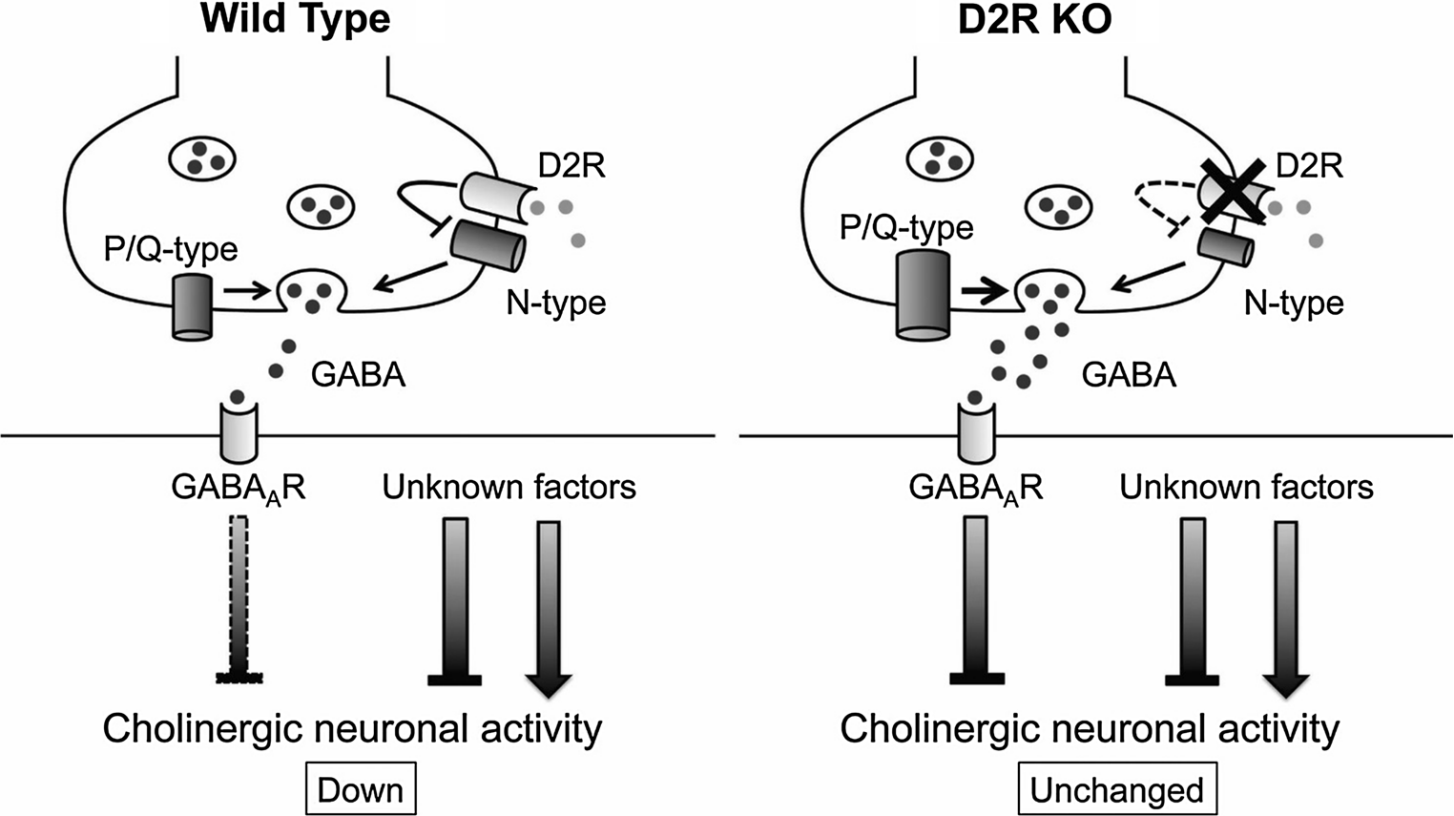
Schematic drawings of a gamma-aminobutyric acid-ergic (GABAergic) synapse onto a striatal cholinergic interneuron in wild type and dopamine D2 receptor knockout (*D2R KO*) mice summarizing current data. *Left* Hypothesized localization of N- and P/Q-type calcium channels as well as of D2R in wild-type mice. Pharmacological results using selective blockers suggest the possibility that P/Q-type calcium channels are localized more closely to the release site than N-type calcium channels, which are coupled to D2R. The* bar below GABA*_*A*_ *R* on the postsynaptic membrane represents the inhibitory effect, with the* width* corresponding to the magnitude of inhibition. *Right* In D2R KO mice, deletion of D2R results in a reduced contribution of N-type calcium channels and an increased contribution of P/Q-type calcium channels. Note the smaller size of the N-type calcium channels in D2R KO mice in the schema and the larger size of the P/Q-type calcium channels, compared with those in wild-type mice. Additional unknown factors should mediate the change in total neuronal activity of cholinergic interneurons

One of the potential neurophysiological events contributing to the BG-related motor control is synaptic transmission in the striatum [18]. In cholinergic interneurons, activation of postsynaptic D1-like receptors depolarizes the membrane by closing potassium channels or opening non-selective cation channels [19], whereas activation of presynaptic D2-like receptors located on GABAergic terminals inhibits GABA release onto cholinergic interneurons [20, 21] by selectively blocking N-type calcium channels [21], as depicted schematically in Fig. 2. However, the role of physiologically released DA as well as the physiological linkage between DA receptors and calcium channels remain unknown.

In this section, recent findings using the D2R-knockout (D2R-KO) mice are reviewed, showing (1) the effect of stimulus frequency on GABAergic transmission on striatal cholinergic interneurons and on their spontaneous firing in order to determine the physiological role of endogenously released DA and (2) the physiological linkage between dopamine D2R and N-type calcium channels in the modulation of GABA release.

### Frequency-dependent suppression of inhibitory postsynaptic current amplitude

Inhibitory postsynaptic currents (IPSCs) evoked in striatal cholinergic interneurons have been shown to be presynaptically inhibited by bath application of DA or D2-like receptor agonists [21, 22]. However, the modulatory roles of physiologically released DA in the striatum remain unknown. To address the question, we examined the dependency of the evoked IPSCs on stimulus frequency between 0.2 and 10 Hz. IPSCs evoked in striatal cholinergic interneurons of wild-type mice showed frequency-dependent suppression during sustained stimulation. To clarify the receptors involved in this frequency-dependent suppression of IPSCs, we then examined the effect of sulpiride, a D2-like receptor antagonist, on the frequency-dependent inhibition of IPSCs in wild-type mice. There was a significant difference (*P* < 0.05) in the amplitude of IPSCs evoked at 5 and 10 Hz in the absence or presence of sulpiride. Based on these results, receptor KO mice would provide a more specific model than pharmacological manipulation when the aim is to identify the receptor subtypes involved. Frequency-dependent suppression of IPSCs examined in DA D2R-KO mice was reduced, and the reduction was distinctly larger than that observed in wild-type mice in the presence of sulpiride, with suppression in D2R-KO mice being significantly (*P* < 0.05) different from that in wild-type mice at each of the corresponding stimulus frequencies.

### High-frequency stimulation-induces inhibition of spontaneous firing

The results of these frequency-dependent suppression experiments suggest that endogenous release of DA with high-frequency stimulation might be involved in this suppression. Therefore, using a cell-attached recording technique with a K-gluconate pipette solution, we examined the effects of high-frequency stimulation (5 and 10 Hz) that mimic the spontaneous firing rate of midbrain dopaminergic neurons [23] on the spontaneous firing of cholinergic interneurons. The spontaneous firing rate in wild-type mice and D2R-KO mice was 5.67 ± 0.94 (*n* = 6) and 5.13 ± 0.26 Hz (*n* = 6), respectively. In wild-type mice, the firing rate was reduced to 3.45 ± 0.64 and 2.85 ± 0.57 Hz (each *n* = 6) after 5 and 10 Hz stimulation, respectively. The firing rate in the presence of sulpiride was not significantly different (*P* > 0.05) from that of a control. Sulpiride antagonized the high-frequency stimulation-induced inhibition of firing. The spontaneous firing rate of cholinergic interneurons in D2R-KO mice was not significantly different (*P* > 0.05) from that of those in wild-type mice. In D2R-KO mice, stimulation with 5 and 10 Hz had no significant (*P* > 0.05) effect on the firing rate.

### Calcium channel subtypes involved in the transmission

Based on the findings of selective coupling between D2-like receptors and N-type calcium channels observed in rats or wild-type mice [21, 22], we examined the effect of D2R deletion on the contribution of calcium channel subtypes to the GABAergic transmission onto striatal cholinergic interneurons using D2R-KO mice. The inhibitory effect of ω-conotoxin (ω-CgTX) on the IPSCs in D2R-KO mice was significantly (*P* < 0.05) smaller than that in wild-type mice. On the other hand, the ω-agatoxin (ω-Aga-TK)-induced inhibitory effect was significantly (*P* < 0.05) larger in D2R-KO mice than in wild-type mice. These findings suggest that there is a tight physiological coupling between D2R and N-type calcium channels in the regulation of GABA release onto striatal cholinergic neurons (Fig. 2).

The selective coupling was further confirmed by the data on developmental changes in ω-CgTX-induced effect in wild-type and D2R-KO mice. It has been reported that in the striatum of rats ω-CgTX-induced inhibition of IPSCs declines with age in the same GABAergic synapse as that examined in the present study [24]. In adult (postnatal days 35–44) wild-type mice, ω-CgTX-induced inhibition of IPSCs was significantly (*P* < 0.05) smaller than that in the young (postnatal days 20–23) wild-type mice. In contrast, in D2R-KO mice, ω-CgTX-induced inhibition of IPSCs was not significantly (*P* > 0.05) different from that in young D2R-KO mice.

### Conclusion

Physiologically released DA in the striatum modulates both GABAergic synaptic transmission onto striatal cholinergic interneurons and the firing of these neurons. The coupling between D2 receptors and N-type calcium channels is tight with respect to release of GABA onto cholinergic interneurons during development (Fig. 2). Further studies are necessary to improve our understanding of the physiological roles of DA and DA receptors in the regulation of total motor control.

## Control of behavioral flexibility by striatal cholinergic interneurons (Kazuto Kobayashi)

The flexible switching of behaviors in response to changes in environment is essential for the survival of animals. This behavioral flexibility is mediated through the neural circuitry linking the prefrontal cortex and basal ganglia [25, 26]. Severe deficits in cognitive flexibility are associated with certain neuropsychiatric diseases, such as schizophrenia and attention deficit hyperactivity disorder [27, 28]. Cholinergic interneurons in the striatum, known as tonically active neurons, respond to a variety of stimuli related to reward prediction, attention and context recognition during learning processes [29, 30]. ACh efflux in the striatum increases in the phase of behavioral switching [31]. However, the exact role of striatal cholinergic interneurons in behavioral flexibility remains uncertain due to complexity of drug dose responses and the broad spectrum of drug affinity to the receptors.

### Roles of striatal cholinergic interneurons in behavioral flexibility

First, we addressed the role of striatal cholinergic interneurons in reversal and extinction learning based on place discrimination by inducing selective elimination of this interneuronal type in rats with immunotoxin (IT)-mediated cell targeting [32]. Transgenic rats were generated that express the human interleukin-2 receptor α-subunit fused to a variant of yellow fluorescent protein under the control of the choline acetyltransferase gene. Injection of a recombinant IT into the dorsal striatum resulted in a selective elimination of cholinergic interneurons, with normal persistence of other neuronal types, such as medium spiny neurons and GABAergic interneurons. Transgenic mice lacking cholinergic interneurons showed the normal acquisition of place discrimination in the modified T-maze but an enhancement of the reversal learning performance of this discrimination. Elimination of cholinergic interneurons from the dorsomedial striatum (DMS), but not from the dorsolateral striatum, was evident in enhanced reversal learning. The transgenic rats lacking cholinergic interneurons in the DMS also exhibited enhancement of extinction learning of place discrimination. These data suggest that striatal cholinergic interneurons in the DMS play an important role in the suppression of behavioral switching, including reversal and extinction learning. It would appear that enhancement of extinction learning in the transgenic rats leads to a promotion of performance in the reversal learning phase.

### The M4 muscarinic receptor mediates suppression of behavioral flexibility

Next we attempted to identify the muscarinic receptor subtypes in the striatum that are involved in behavioral switching by using gene-specific silencing of the M_1_ and M_4_ muscarinic receptors [32]. Lentiviral vector encoding the short-hairpin RNA for M_1_ or M_4_ receptor was injected into the DMS of the rats, resulting in a significant reduction of the mRNA levels corresponding to the receptor subtypes. Gene silencing of the M_4_ muscarinic receptor enhanced the place reversal learning in a manner similar to the performance observed in transgenic rats lacking DMS cholinergic interneurons. In contrast, gene silencing of the M_1_ muscarinic receptor did not affect the performance of reversal learning. These data suggest that behavioral flexibility is mainly mediated through the M_4_ but not the M_1_ muscarinic receptor in the DMS.

### Conclusion

**Fig. 3:**
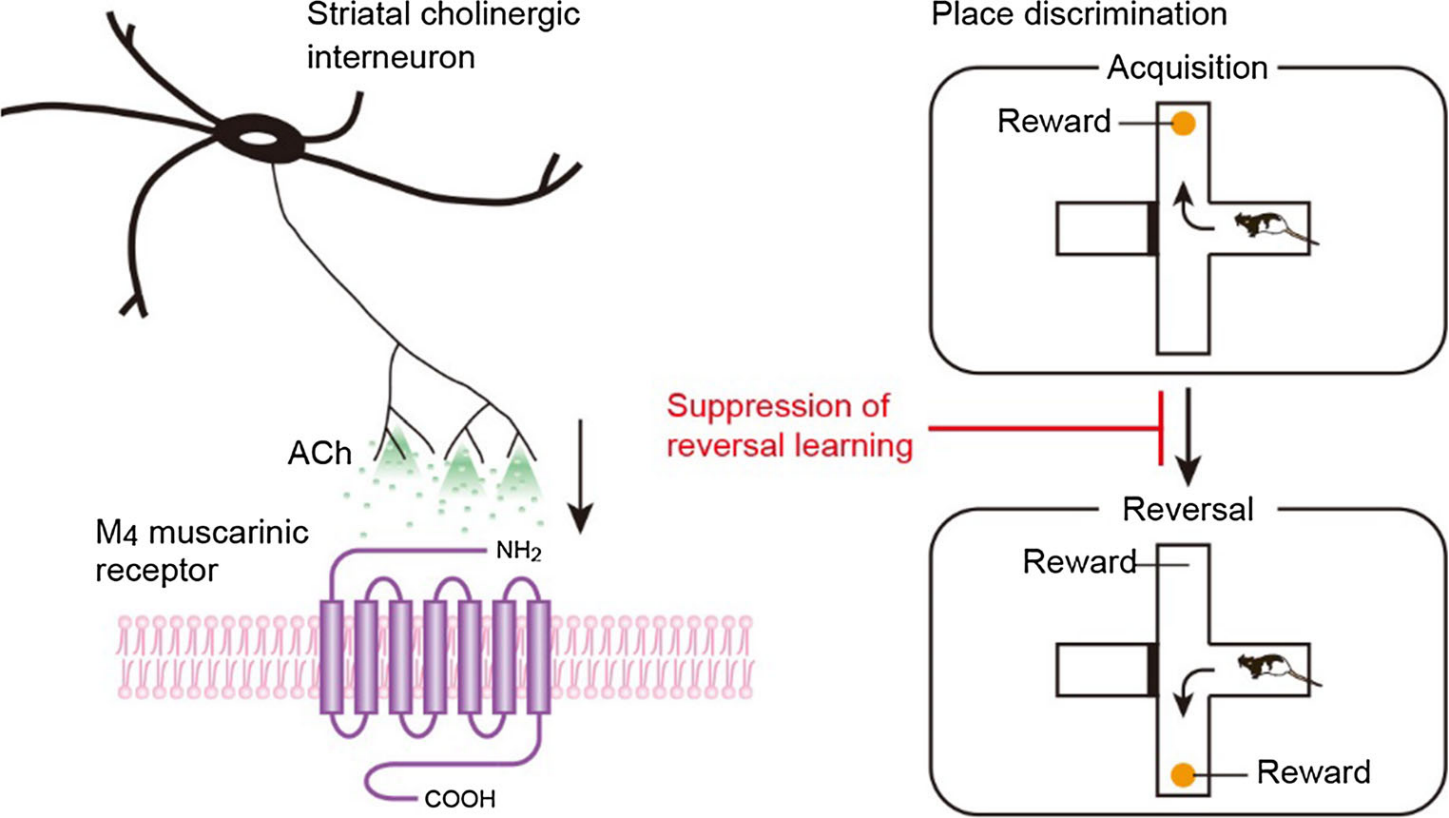
Striatal cholinergic interneurons suppress behavioral flexibility via the M_4_ muscarinic receptor. Activation of striatal cholinergic interneurons increases acetylcholine (*ACh*) release and stimulates M_4_ muscarinic receptor, resulting in the suppression of behavioral switching in response to changed contingency

Based on these results, we conclude that striatal cholinergic interneurons in the DMS act to inhibit behavioral flexibility and that this action is predominantly mediated through the M_4_ muscarinic receptor [32] (summarized in Fig. 3). Striatal cholinergic interneurons may regulate the neural circuitry linking the prefrontal cortex and DMS to suppress the information processing that is involved in flexible switching of the behavior in response to changes in the environment. Our expectation is that the mechanism underlying how the M_4_ receptor signaling controls behavioral switching will be elucidated.

## Regulation of phosphorylation-states of DARPP-32 by protein phosphatases (Akinori Nishi)

**Fig. 4:**
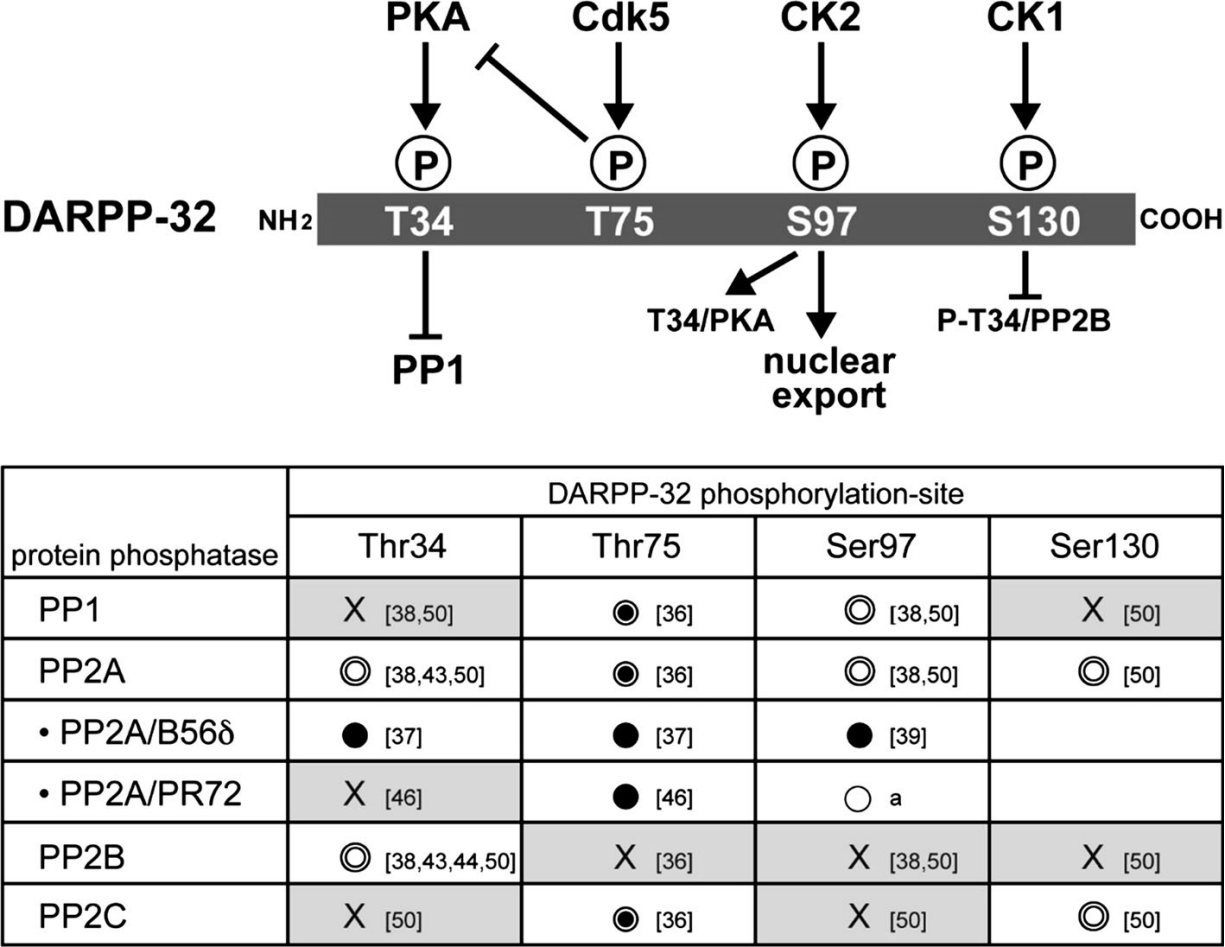
Protein phosphatases involved in dephosphorylation of dopamine- and cAMP-regulated phosphoprotein, M_*r*_ 32,000 (*DARPP-32*) at four sites [*T34* (Thr34),* T75* (Thr75),* S97* (Ser97),* S130* (Ser130)].* Ser* serine,* Thr* threonine. The protein phosphatases for each site were determined using various preparations of protein phosphatases:  Purified protein phosphatases,  protein phosphatases in striatal homogenate,  protein phosphatases expressed in cells,  protein phosphatase inhibitors; *X* No dephosphorylation. *a* Preliminary slice data (unpublished observations, A. Nishi),* numbers in square brackets* References in reference list. *PKA* Protein kinase A,* Cdk 5* cyclin-dependent kinase 5,* CK1*,* -2* casein kinase1, 2,* PP* protein phosphatase

Dopamine- and cAMP-regulated phosphoprotein, M_*r*_ 32,000 (DARPP-32) is an essential regulator of DA signaling in striatal medium spiny neurons [33]. DA signaling is regulated by the phosphorylation states of DARPP-32 at four major sites in both D1-type/striatonigral and D2-type/striatopallidal neurons [34]. The most important phosphorylation site of DARPP-32 is Thr34 where DARPP-32 is phosphorylated by protein kinase A (PKA), resulting in its conversion into a potent inhibitor of protein phosphatase-1 (PP1) (Fig. 4) [33]. When PKA is activated, the inhibition of PP1 increases the phosphorylation states of the substrates and the activity of many downstream effectors, including various neurotransmitter receptors, ion channels and transcription factors.

DARPP-32 is also phosphorylated at Thr75 by cyclin-dependent kinase 5 (Cdk5), at Ser97 by casein kinase 2 (CK2) and at Ser130 by casein kinase (CK1) in the mouse sequence (Fig. 4). DARPP-32 phosphorylated at Thr75 by Cdk5 inhibits PKA activity and therefore suppresses DA D1R signaling [35]. DA, by sequentially activating D1R, PKA and protein phosphatase 2A (PP2A)/B56δ subunit of PP2A induces dephosphorylation of DARPP-32 at Thr75, resulting in de-inhibition of PKA [36, 37]. Further activation of PKA and inhibition of PP1 by phospho-Thr34 (P-Thr34) DARPP-32 amplify DA D1R signaling. Phosphorylation of DARPP-32 at Ser97 by CK2 was originally found to facilitate phosphorylation of DARPP-32 at Thr34 by PKA [38]. In addition, the phosphorylation state of DARPP-32 at Ser97 regulates the localization of DARPP-32 in the nucleus. Phospho-Ser97 (P-Ser97) functions as a nuclear export signal of DARPP-32, and dephosphorylation of DARPP-32 at Ser97 by PKA-activated PP2A/B56δ results in the nuclear accumulation of P-Thr34 DARPP-32, leading to inhibition of nuclear PP1, phosphorylation of histone H3 and increased gene expression [39]. DARPP-32 is also phosphorylated at Ser130 by CK1 [40]. Phosphorylation of Ser130 by CK1 inhibits dephosphorylation of Thr34 by PP2B [41].

### Desphosphorylation of DARPP-32 by protein phosphatases

The phosphorylation state of DARPP-32 at each site is determined by the balance between phosphorylation and dephosphorylation [33]. Dephosphorylation of DARPP-32 at the four major sites is regulated by the PPP (PP1, PP2A and PP2B) and PPM (PP2C) families of Ser/Thr protein phosphatases (Fig. 4) [42].

#### Thr34 dephosphorylation

 P-Thr34 DARPP-32 (PKA site) is dephosphorylated by PP2B (calcineurin) and PP2A in vitro [43, 44]. As PP2B is activated by Ca^2+^, glutamate via activation of the NMDA/AMPA receptor/Ca^2+^, signaling induces Thr34 dephosphorylation by PP2B, resulting in de-inhibition of PP1. In this regard, DA/D1R/PKA and glutamate/NMDA/AMPA receptor signaling can mutually counteract each other[45]. Two heterotrimeric forms of PP2A, the PKA-sensitive PP2A/B56δ [37] and Ca^2+^-sensitive PP2A/PR72 [46], are involved in the dephosphorylation of DARPP-32. P-Thr34 DARPP-32 is a substrate for PP2A/B56δ, but not for PP2A/PR72 [37], and the activation of PP2A/B56δ by PKA may result in the turning off of DA/D1R/PKA/P-Thr34 DARPP-32 signaling. It is likely that both PP2B and PP2A/B56δ contribute to maintenance of the basal level of P-Thr34 DARPP-32 because inhibition of PP2B by cyclosporin A and inhibition of PP2A by okadaic acid in striatal slices synergistically increase the level of P-Thr34 DARPP-32 [47].

#### Thr75 dephosphorylation

 P-Thr75 DARPP-32 (Cdk5 site) is dephosphorylated mainly by PP2A and to a lesser extent by PP1 and PP2C in vitro [36, 48]. Two types of PP2A, namely, PP2A/B56δ and PP2A/PR72, dephosphorylate P-Thr75 DARPP-32 following activation of PKA and Ca^2+^ signaling, respectively [36, 37, 46, 48]. PP2A/B56δ by PKA removes the inhibition of PKA by P-Thr75 DARPP-32 as a positive-feedback loop [36, 37, 48].

#### Ser97 dephosphorylation

 P-Ser97 DARPP-32 (Ck2 site) is dephosphorylated by PP2A and PP1 in vitro [38]. Activation of DA/D1R/PKA signaling induces dephosphorylation of DARPP-32 at Ser97 by PKA-activated PP2A/B56δ, leading to nuclear localization of DARPP-32 [39, 49]. Preliminary slice data reveal that Ca^2+^-activated PP2A/PR72 also dephosphorylates P-Ser97 DARPP-32 (unpublished observations, A. Nishi), suggesting that both DA and glutamate signals induce nuclear localization of DARPP-32. The role of PP1 in the dephosphorylation of P-Ser97 DARPP-32 has not yet been characterized.

#### Ser130 dephosphorylation

 P-Ser130 DARPP-32 (CK1 site) is dephosphorylated by PP2A and PP2C in vitro [50]. The role of CK1 in Ser130 phosphorylation has been implicated in the action of mGluR1/5 receptors [51, 52] and psychostimulants [53]. However, the physiological roles of PP2A or PP2C in Ser130 dephosphorylation have not yet been elucidated.

### DA and glutamate signaling mediated by dephosphorylation of DARPP-32

Activation of PKA by DA/D1R signaling induces the phosphorylation of DARPP-32 at Thr34 and the activation of PP2A/B56δ-mediated feedback loops, resulting in (1) increased dephosphorylation of P-Thr34 DARPP-32 (negative feedback), (2) decreased inhibition of PKA due to dephosphorylation of P-Thr75 DARPP-32 (positive feedback) and (3) decreased efficacy of Thr34 phosphorylation by PKA due to dephosphorylation of Ser97 DARPP-32 (negative feedback).

Activation of the glutamate/NMDA/AMPA receptor/Ca^2+^ signaling increases the activities of PP2B and PP2A/PR72. Activated PP2B dephosphorylates P-Thr34 DARPP-32, whereas activated PP2A/PR72 dephosphorylates P-Thr75 and P-Ser97. Dephosphorylation of these two sites affects the level of P-Thr34 DARPP-32 via two mechanisms: decreased inhibition of PKA by P-Thr75 DARPP-32 and decreased efficacy of Thr34 phosphorylation by PKA.

It has been proposed that DA via activation of D1R/PKA signaling and glutamate via activation of NMDA/AMPA receptor/Ca^2+^/PP2B signaling counteract each other in the regulation of DARPP-32 phosphorylation at Thr34 [54, 55]. However, the level of P-Thr34 DARPP-32 is also regulated by other complex pathways. Two types of PP2A, namely, the PKA-sensitive PP2A/B56δ and Ca^2+^-sensitive PP2A/PR72, also play roles in the regulation of Thr34 phosphorylation via direct and DARPP-32-mediated indirect mechanisms.

## Physiological perspective on deep brain stimulation with optogenetics and closed-loop control for ameliorating parkinsonism [Susumu Takahashi (corresponding author of section), Fuyuki Karube and Fumino Fujiyama]

Parkinson disease is a neurodegenerative movement disorder in which dopaminergic (DAergic) cells in the SNc are progressively lost. One suggested cause of PD is that the loss of DAergic inputs to the striatum induce a malfunctioning of the BG circuitry. The treatment of PD symptoms has been traditionally divided into three strategies. In the historical context, PD symptoms have been treated through surgical removal of the internal segment of the globus pallidus (GPi), subthalamus or thalamus. This is a high-risk treatment, and it is unpredictable whether such irreversible treatment will result in severe damage to the functioning of the BG. A second strategy is DA replacement therapy. Levodopa is the most common drug used in this context, with the aim to replace the diminished supply of DA in the brain, thereby ameliorating PD symptoms. However, prolonged use of levodopa and other drugs used for this purpose can cause side-effects in PD patients, including dyskinesia, probably due to the effect of the supplemented dopamine in influencing the neuronal state of unwanted brain regions and/or cell types, such as serotonergic cells [56]. Deep brain stimulation (DBS) also ameliorates PD symptoms by stimulating a part of the BG circuitry at a high frequency rate (approx. 120 Hz) irrespective of the current brain state. Electrodes inserted into GPi, subthalamic nucleus (STH) or thalamus effectively improve the parkinsonism by the high-frequency stimulations, whereas low-frequency stimulations worsen the symptoms. It is still unknown whether DBS activates or inactivates the target neuronal tissues. On the other hand, a classical model of the BG circuitry [57] contains two pathways: a direct pathway from the striatum to GPi or SNr and an indirect pathway from the striatum to the GPi or SNr via the external segment of the globus pallidus (GPe) and/or the STH. In PD patients, the loss of DAergic cells must cause dysfunction in both pathways. Indeed, both the firing rates and power of the beta band frequency in local field potentials (LFPs) of the BG circuitry have been found to increase pathologically with increasing progress of PD symptoms, indicating that the pathways or their microcircuits in the BG circuitry malfunction as a whole. Surgical removal treatment and DBS may prevent the irregular information flow into the pathways, while long-term replacement therapy may create unnatural pathways in the BG circuitry. However, the exact cause of PD remains unknown. A recent cutting-edge technology, termed optogenetics, enables the manipulation of the activity of genetically engineered neurons at a high temporal resolution by photostimulations. Alternatively, an engineering technique, termed closed-loop control, is being incorporated into the DBS system. The closed-loop DBS can provide interventions only when required by detecting abnormal neurological signals. Such technical advancements should shed light on the cause of PD. In the following sections we review and discuss the cause of PD beyond the loss of dopaminergic neurons based on physiological evidence reported using the DBS system with optogenetic technology and closed-loop control.

### Optogenetic DBS

Since the electrical effect of DBS is widely spread over various cell types around the target regions, the stimulation influences not only excitatory cells and inhibitory interneurons but also glial cells, although, as mentioned, whether DBS activates or inactivates the target neuronal tissue is as yet unknown. Cell-type-specific activation or inactivation with light-sensitive proteins, called optogenetic technology, has recently been used to elucidate the effects of DBS. In response to photostimulation, a light-activated cation channel, termed channelrhodopsin (ChR), is able to activate excitatory channels to increase the firing rate of the expressing neurons; a light-activated chloride pump, termed halorhodopsin (HR), is able to activate inhibitory pumps and channels to inhibit the firing of expressing neurons. Together with Cre-lox recombination, those engineered proteins, called opsins, can be expressed in a specific cell type. Thus, cell type-specific control can be achievable using optogenetic technology.

Kravitz and colleagues elucidated pathway-dependent therapeutic effects on parkinsonism in mice using optogenetics technology [58]. In the 6-hydroxydopamine (OHDA) lesion mouse model of PD, for activation of only the direct pathway of the BG circuitry, ChR2 expression was required in medium spiny neurons (MSNs) containing D1R in the striatum of the mice. The light activation of direct pathway neurons improved bradykinesia. To only activate the indirect pathway, ChR2 was expressed in MSNs containing D2R in the striatum of mice with normal motor behavior. Surprisingly, the activation of indirect pathway neurons generated parkinsonism. It is well known that the loss of DAergic cells disrupts the function which facilitates activity of the direct pathway neurons and suppresses activity of the indirect pathway neurons. These results provide two insights into the cause of PD. First, optogenetic pathway-specific activation can compensate for the functioning of DAergic inputs to the striatum, thereby ameliorating parkinsonism; second, suppression of the activity of indirect pathway neurons appears to be one of the main causes of PD.

Deisseroth and colleagues precisely applied such selective expression of light-sensitive proteins to cortico–BG circuitry to reveal the cause of the electrical DBS of the STH (STH-DBS) [59]. Regarding the question of why electrical STH-DBS ameliorates parkinsonism, these authors proposed that STH-DBS facilitates or suppresses the STH neurons per se or the afferent fibers into the STH. Photostimulation of the STH neurons expressing ChR2 or HR under the Ca^2+^/calmodulin-dependent protein kinase IIα (CaMKIIα) promoter did not ameliorate parkinsonism in a 6-OHDA model of rats, suggesting that both the facilitation and suppression of the STH firing do not have a direct effect on the amelioration of parkinsonism. Photostimulation of only afferent fibers arriving from layer V of the motor cortex using transgenic mice expressing ChR2 under the Thy1 promoter has been shown to improve parkinsonism. These results suggest that the beneficial anatomical target of the STH-DBS is afferent fibers—i.e. the hyperdirect pathway [60]—from layer V of the motor cortex to the STH. In other words, the STH neurons alone do not appear to play a crucial role in the DBS treatment. These results also enable a novel interpretation that the pyramidal tract neurons in layer V of the motor cortex and/or the downstream structures are the actual target of the DBS therapy.

### Closed-loop DBS

In a control theory of engineering fields, the traditional DBS is referred to as an open-loop system because stimulations are constantly applied irrespective of the pathological brain state. Closed-loop systems incorporating feedback between input and output signals should effectively control the pathological brain state. However, there are many technical issues associated with the implementation of the closed-loop system into the DBS, such as (1) the closed-loop system must operate automatically in real time with a submillisecond precision; (2) focal interventions with high temporal precision are necessary to maintain a reliable feedback control; (3) algorithms that can process inputs in a time-dependent manner are needed. Salient pathological signals could be used to serve as inputs. Typically, pathological changes in firing rates or timing are linked to the onset of worsening of parkinsonism [61], suggesting that such pathological firing patterns could potentially be used to trigger intervention in the closed-loop system. In PD patients, pathological beta oscillations shown in LFPs can be used as inputs to trigger the focal stimulation. Currently, taking into consideration clinical efficacy, the electrical DBS is an ideal effector to deliver interventions to the target. The closed-loop DBS can offer increased efficacy and clinical benefits because it enables intermittent stimulations, decreases tissue damage and reduces battery consumption. The therapeutic effect of the traditional open-loop DBS disappear immediately after the stimulation is turned off. In contrast, the closed-loop DBS ideally reverts from pathological activity patterns of the BG circuitry to normal physiological patterns. Moreover, as theoretical studies have postulated [62], the closed-loop DBS incorporating the plasticity mechanism may maintain the therapeutic effect after the operation.

Bergman and colleagues examined the efficacy of their closed-loop DBS system with a unique real-time feedback device [63]. They inserted recording electrodes into the motor cortex and GPi in the BG circuitry in a 1-methyl-4-phenyl-1,2,3,6-tetrahydropyridine (MPTP)-induced PD model of nonhuman primates and then recorded spiking activity in the motor cortex or GPi as reference signals for triggering the system, with the target of the DBS being the GPi. When the spikes in the motor cortex triggered the system, the improvement of parkinsonism was more efficient than that of the traditional open-loop DBS. At the same time, the pathological enhancement of GPi oscillatory activity was prominently reduced. In contrast, when the spikes in the GPi triggered the system, parkinsonism worsened, together with increased GPi oscillatory activity. These results suggest that the enhanced oscillatory activity was tightly linked to the pathophysiology of PD. Thus, the results of this study strongly support the view that the closed-loop approach is the most efficient for DBS aimed at ameliorating parkinsonism.

### Discussion and conclusion

**Fig. 5:**
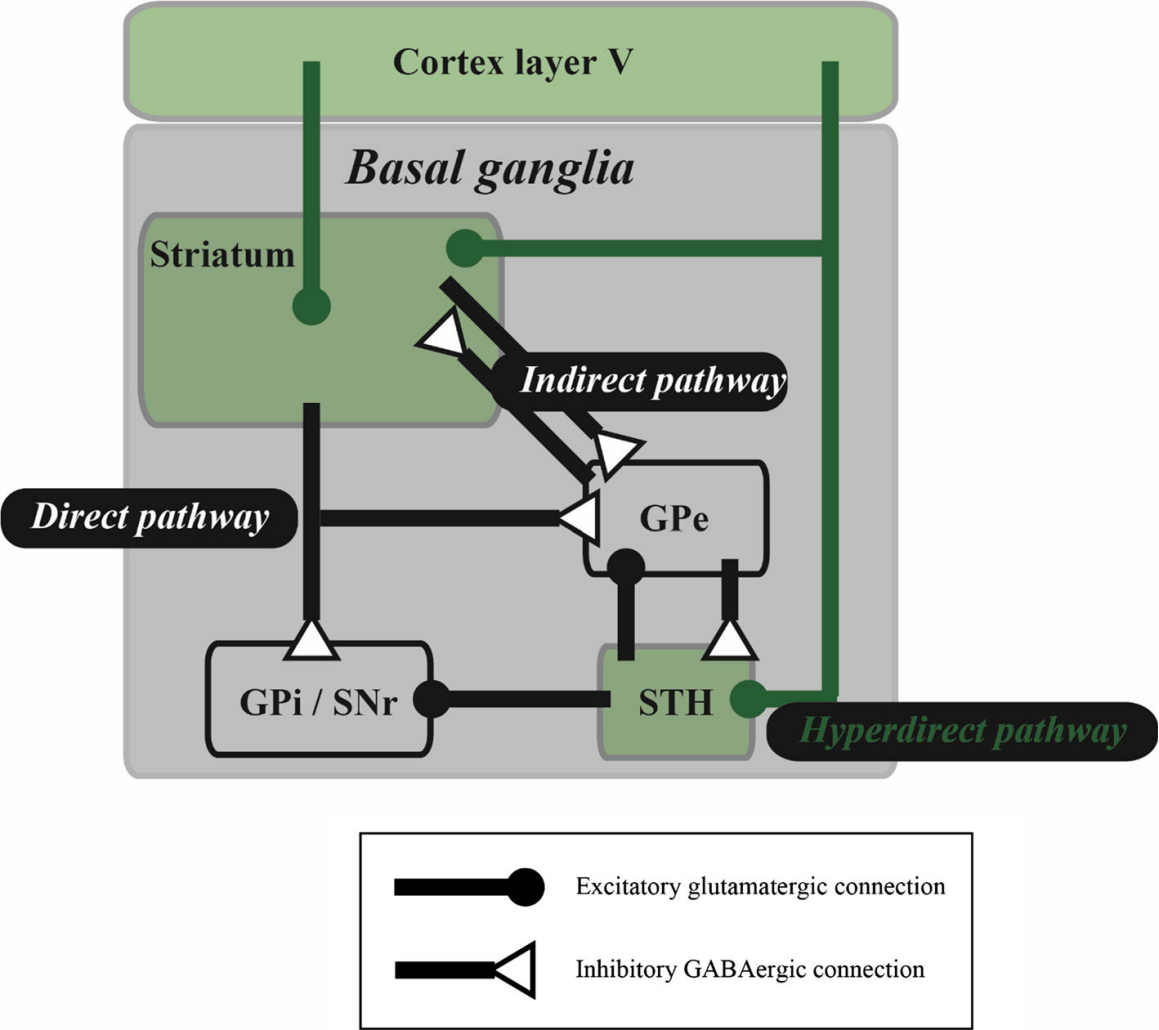
Efficient pathway and structure unveiled by deep brain stimulation (DBS) with optogenetics and closed-loop control. Selected anatomical connections are illustrated in a simplified cortico–basal ganglia circuitry. Optogenetic selective activation revealed that unlike the indirect pathway, activation of medium spiny neurons expressing the dopamine D1 receptor—i.e. direct pathway—in the striatum [3] and high-frequency stimulations to fibers from cortex layer V to subthalamic nucleus (*STH*)—i.e. hyperdirect pathway [4]—ameliorate parkinsonism. In addition, closed-loop stimulations to the internal segment of the globus pallidus (*GPi*), triggered by spiking activity of the motor cortex, are efficient for improving parkinsonism [8]. These reports suggest that the hyperdirect pathway (*green lines*) alone or together with its downstream structures, including cortex layer V, striatum and STH (*green-shaded areas*) play a crucial role in DBS treatment. *GPe* External segment of globus pallidus,* GABA* gamma-aminobutyric acid

Deep brain stimulation together with optogenetic technology and closed-loop control is a promising approach that may open new doors to the exploration of the cause of PD. Indeed, with such technological advances, the physiological examination of PD in animal models has delivered remarkable data on the crucial anatomical structures and pathways causing the PD symptoms beyond the loss of DAergic cells, as shown in Fig. 5. These anatomical structures and pathways are extremely important clues to the elucidation of the cause of PD.

The full potential of such advanced technologies can be applied in studies using animal models because they can be combined with another state-of-the-art technology which simultaneously monitors multiple single-unit activities [64, 65], in contrast to clinical studies. In this same context, optogenetic technology is not currently available in humans, in part due to safety concerns arising from the requirement for gene therapy to achieve opsin expression. In optogenetics, specific opsin genes are introduced into the host via viral vectors. Fortunately, the safety of specific viral vectors has been confirmed for gene therapy for PD, opening the door to optogenetic technology for the treatment of PD in humans. With respect to closed-loop DBS, reliable noninvasive DBS devices for PD have not been developed at the clinical level. Neurostimulation systems, including not only invasive DBS, but also noninvasive transcranial magnetic stimulation (TMS) and transcranial direct current stimulation (tDCS), are now an established therapy for several neurological disorders. For example, repetitive TMS has been shown to provide pain relief and improve the quality of life in patients with neuropathic pain [66]. Moreover, the closed-loop system incorporating epilepsy which effectively stimulates the foci only when it detects early seizures can reduce seizure frequency in a selected patient population [67]. As shown in Fig. 5, the motor cortex may be an effective target for the neurostimulation in ameliorating parkinsonism. Thus, in the future, invasive electrodes for DBS could be replaced with noninvasive TMS or tDCS.

If a system incorporating closed-loop control with optogenetic photostimulation were to be developed, it would achieve not only the temporal specificity of electrical stimulation, but also cell-type specific closed-loop control with excitation and inhibition in response to the pathological brain state. Such a system would help determine the cause of PD in more detail and is expected to offer an ideal combinational therapeutic product for treating several neurological disorders.

## Concluding remarks

In this review we have outlined recent advances in the field of BG circuitries as well as clarified several unsolved and unidentified issues on transmission mechanisms and physiological functions, including the interaction between glial cells and various types of striatal neurons and the physiological release mechanisms of DA. Future studies addressing these unsolved questions might well lead to the development of new therapeutic tactics for BG-related diseases.

## Abbreviations

CaMKII: Ca^2+^/calmodulin-dependent protein kinase II
ChR2: Channelrhodopsin2
DA: Dopamine
D1R (D2R): Dopamine D1 (D2) receptor
DBS: Deep brain stimulation
GPi/GPe: Internal/external segment of globus pallidus
HR: Halorhodopsin
LFP: Local field potential
MSN: Medium spiny neuron
MPTP: 1-Methyl-4-phenyl-1,2,3,6-tetrahydropyridine
OHDA: Hydroxydopamine
PD: Parkinson disease
SNc/SNr: Substantia nigra pars compacta/reticulata
STH: Subthalamic nucleus
tDCS: Transcranial direct current stimulation
TMS: Transcranial magnetic stimulation
